# Supplementary figures and images for: A Survey on the Expression of the Ubiquitin Proteasome System Components HECT- and RBR-E3 Ubiquitin Ligases and E2 Ubiquitin-Conjugating and E1 Ubiquitin-Activating Enzymes during Human Brain Development
Source: Int J Mol Sci. 2024 Feb 17;25(4):2361. doi: 10.3390/ijms25042361 (PMC10889685; doi:10.3390/ijms25042361)

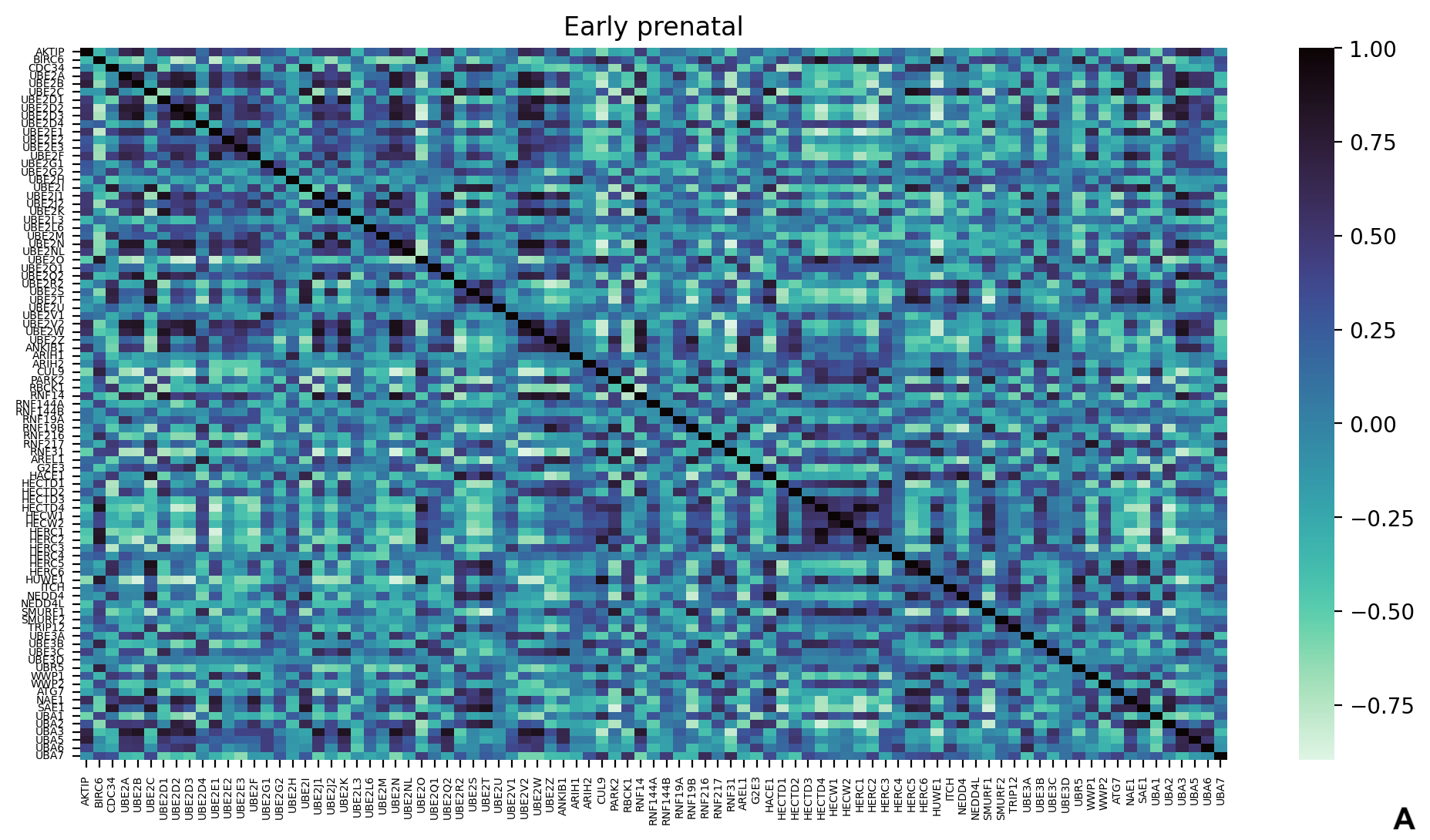

Supplement: Supplementary file 1 [file ijms-25-02361-s001.zip › Figure S1/A.png]

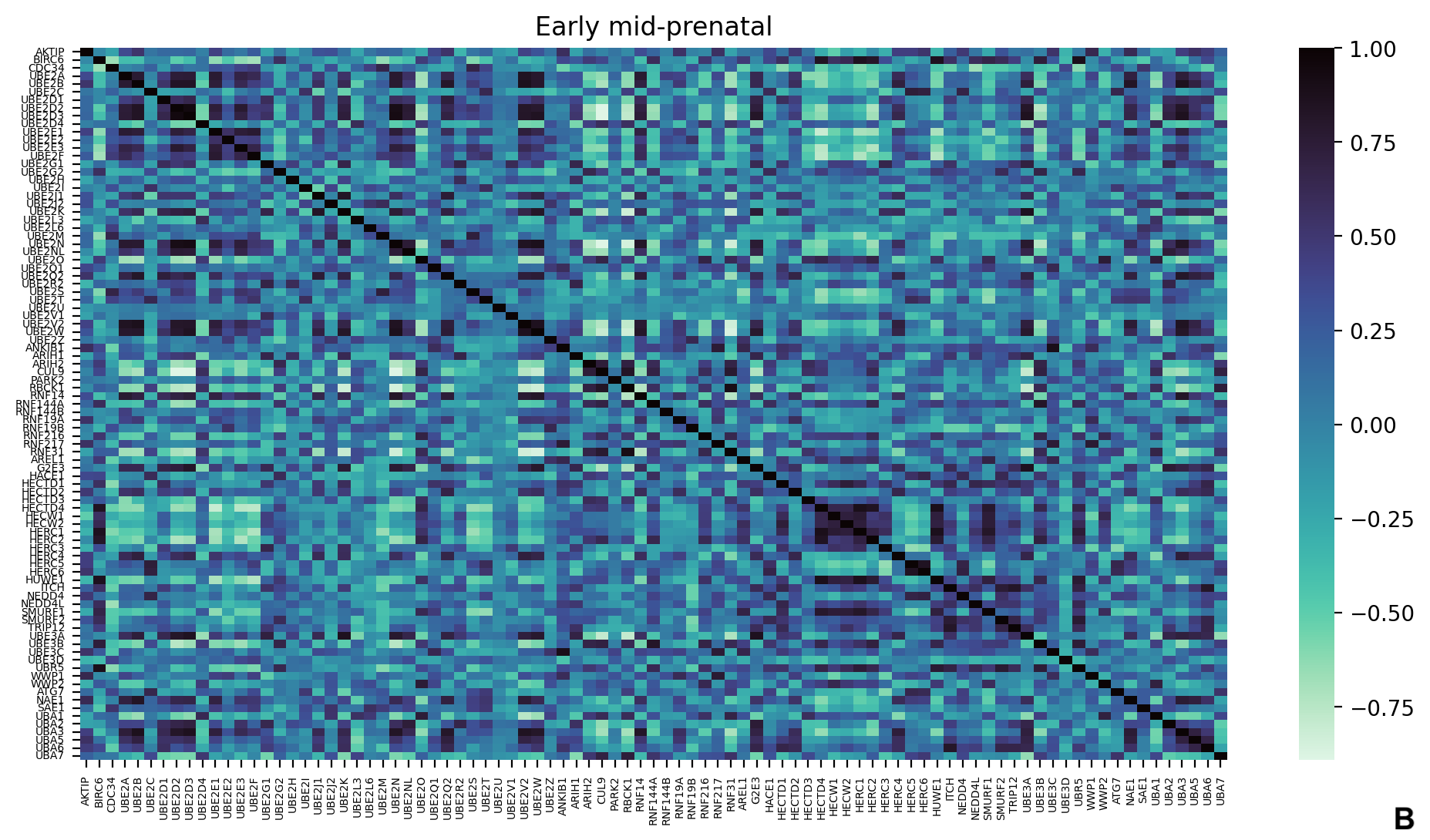

Supplement: Supplementary file 1 [file ijms-25-02361-s001.zip › Figure S1/B.png]

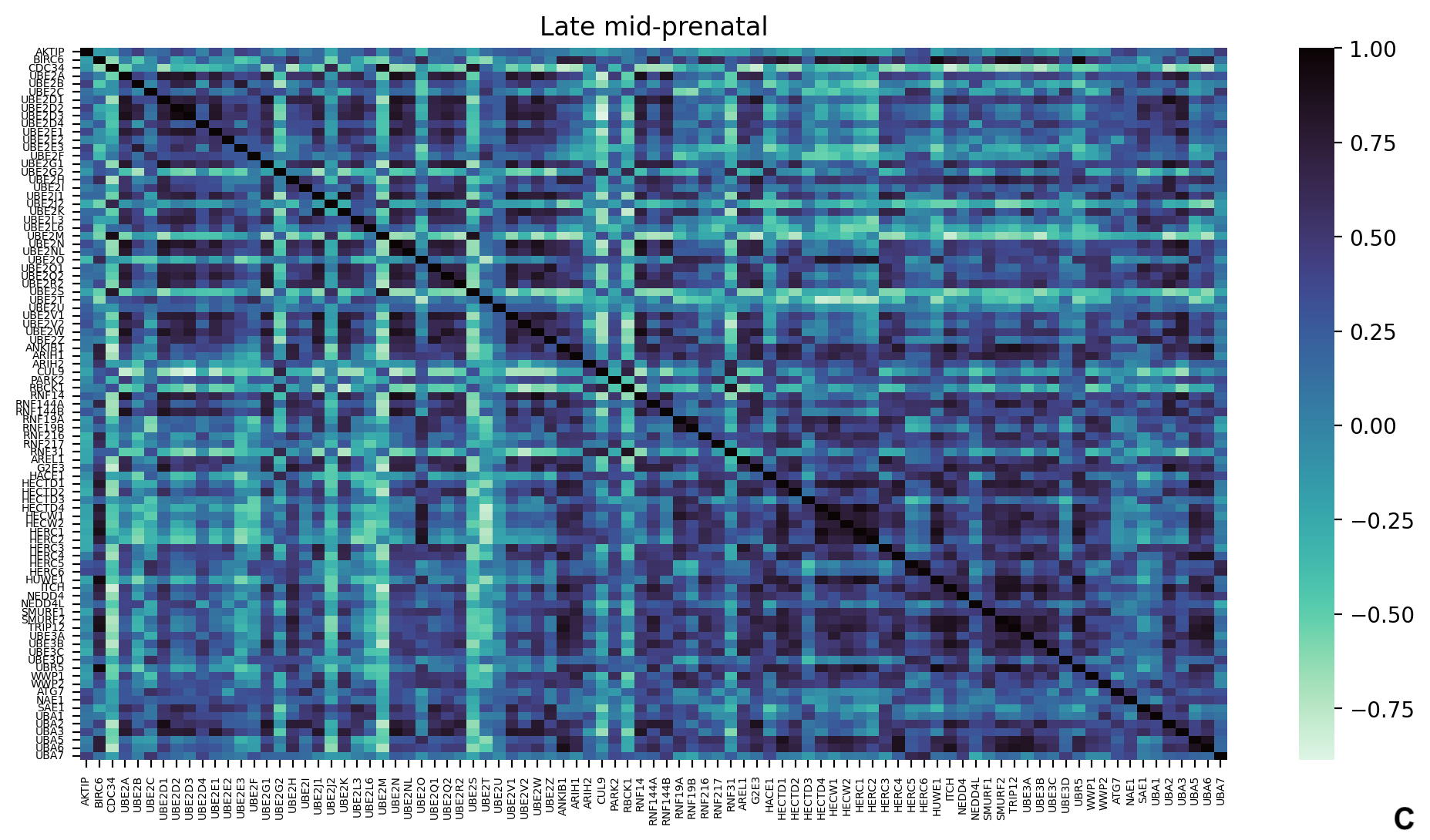

Supplement: Supplementary file 1 [file ijms-25-02361-s001.zip › Figure S1/C.png]

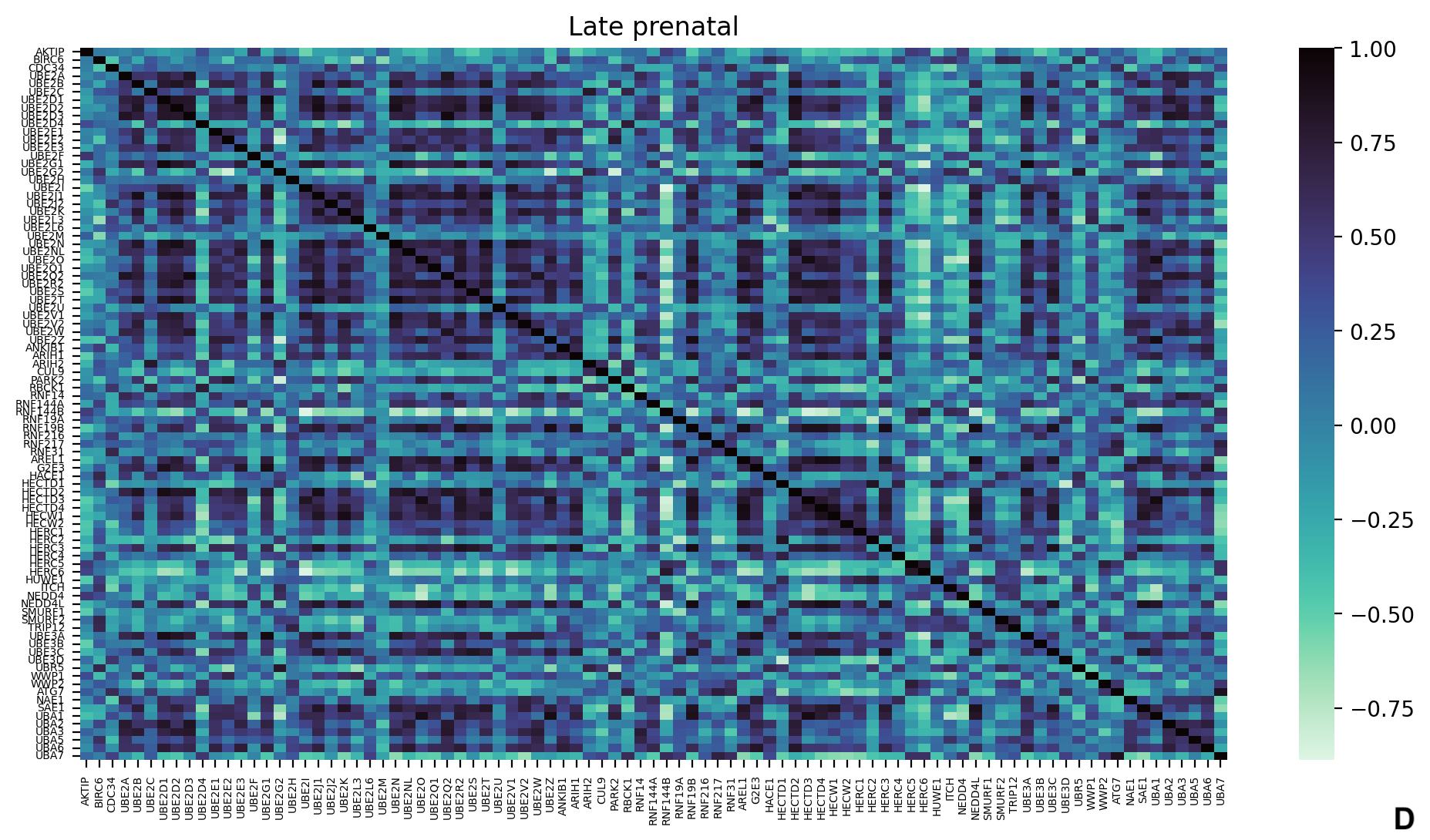

Supplement: Supplementary file 1 [file ijms-25-02361-s001.zip › Figure S1/D.png]

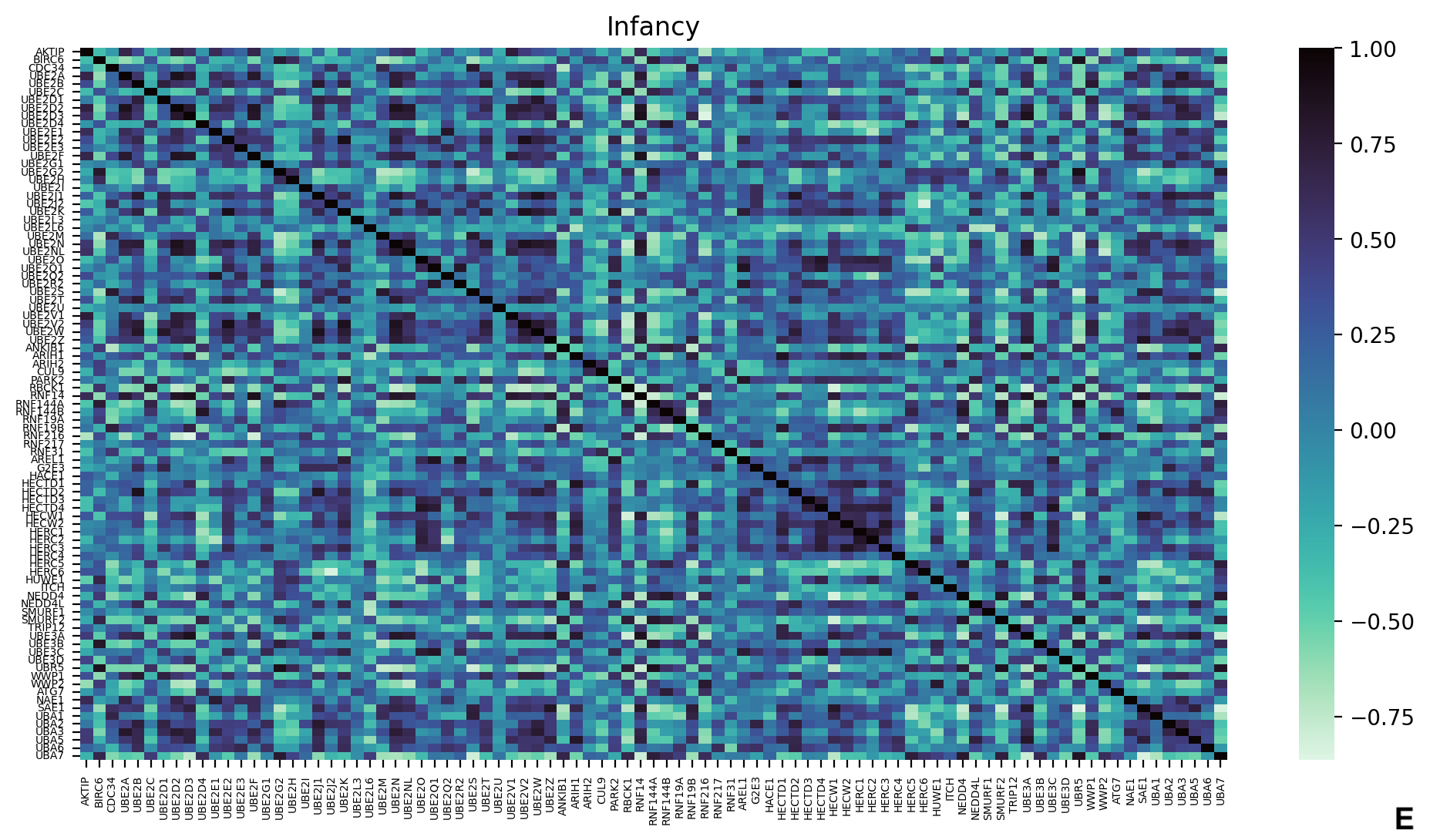

Supplement: Supplementary file 1 [file ijms-25-02361-s001.zip › Figure S1/E.png]

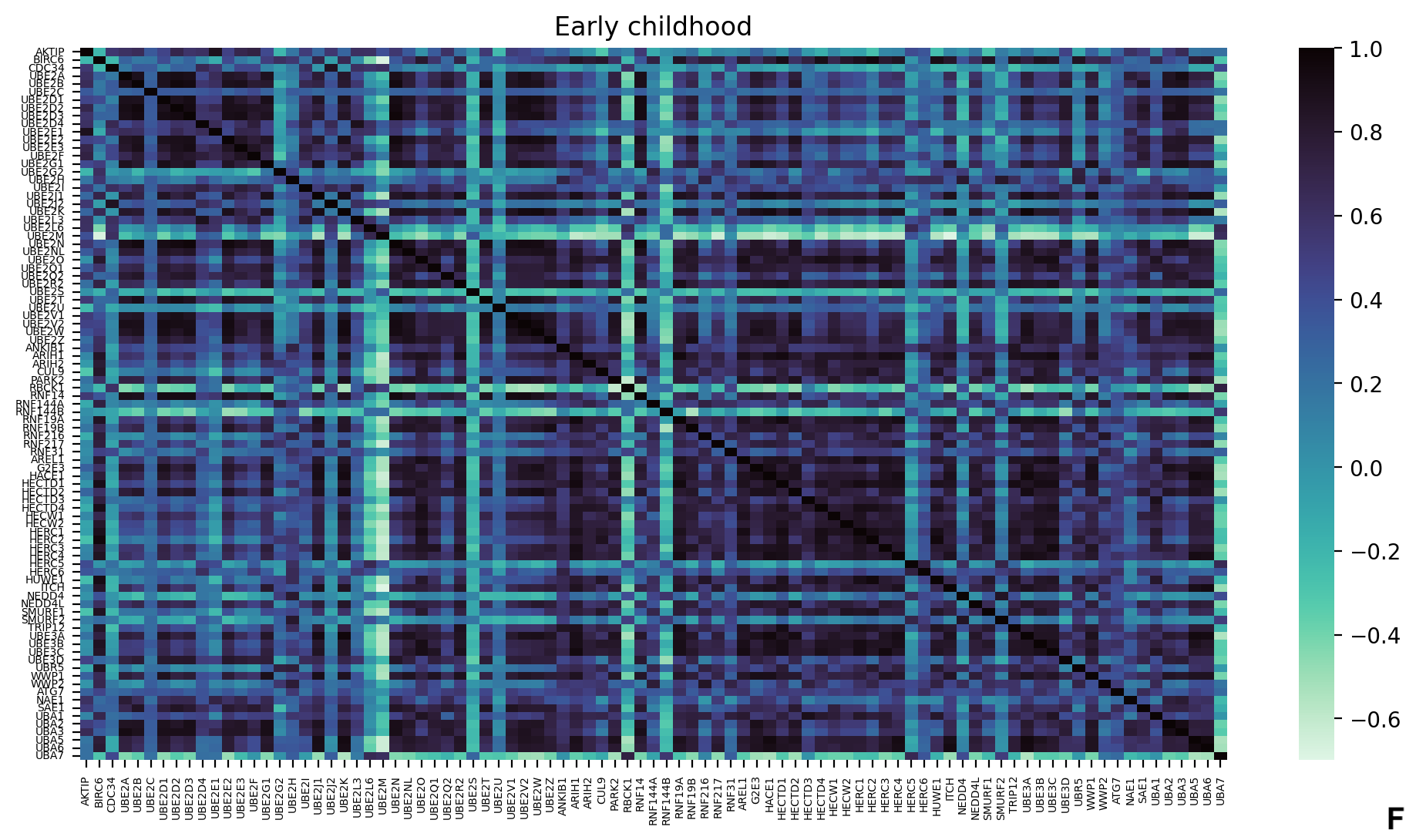

Supplement: Supplementary file 1 [file ijms-25-02361-s001.zip › Figure S1/F.png]

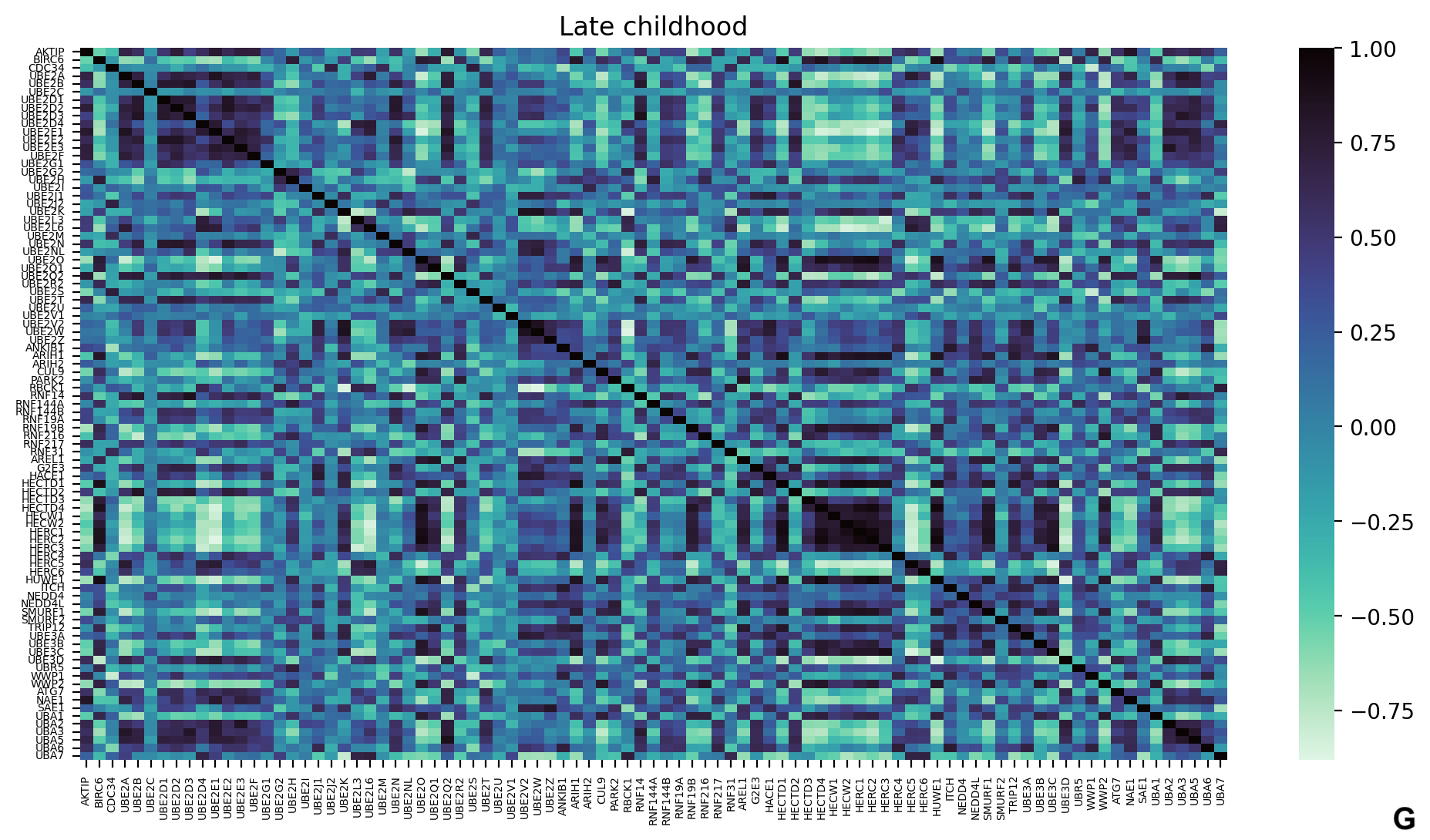

Supplement: Supplementary file 1 [file ijms-25-02361-s001.zip › Figure S1/G.png]

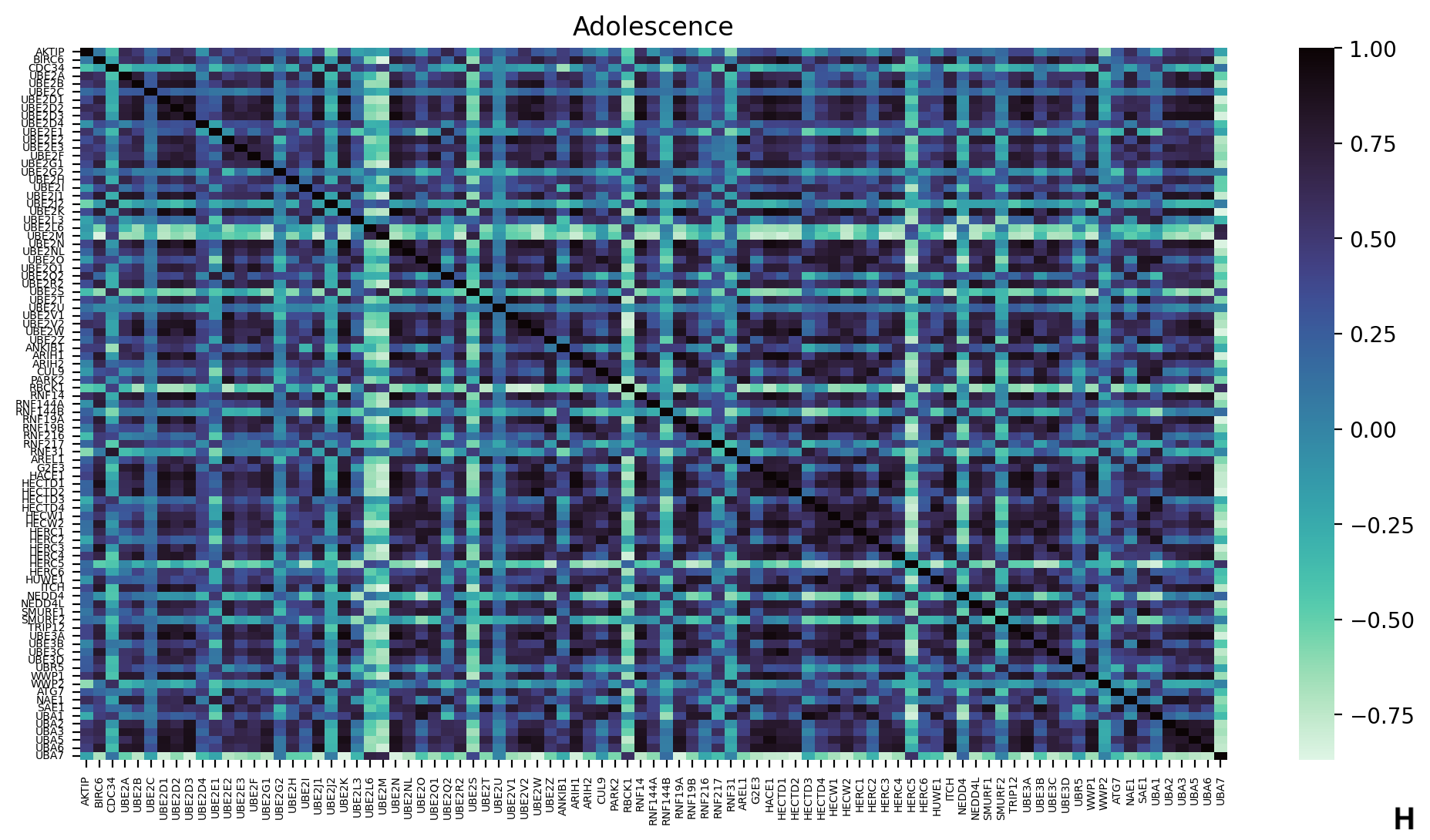

Supplement: Supplementary file 1 [file ijms-25-02361-s001.zip › Figure S1/H.png]

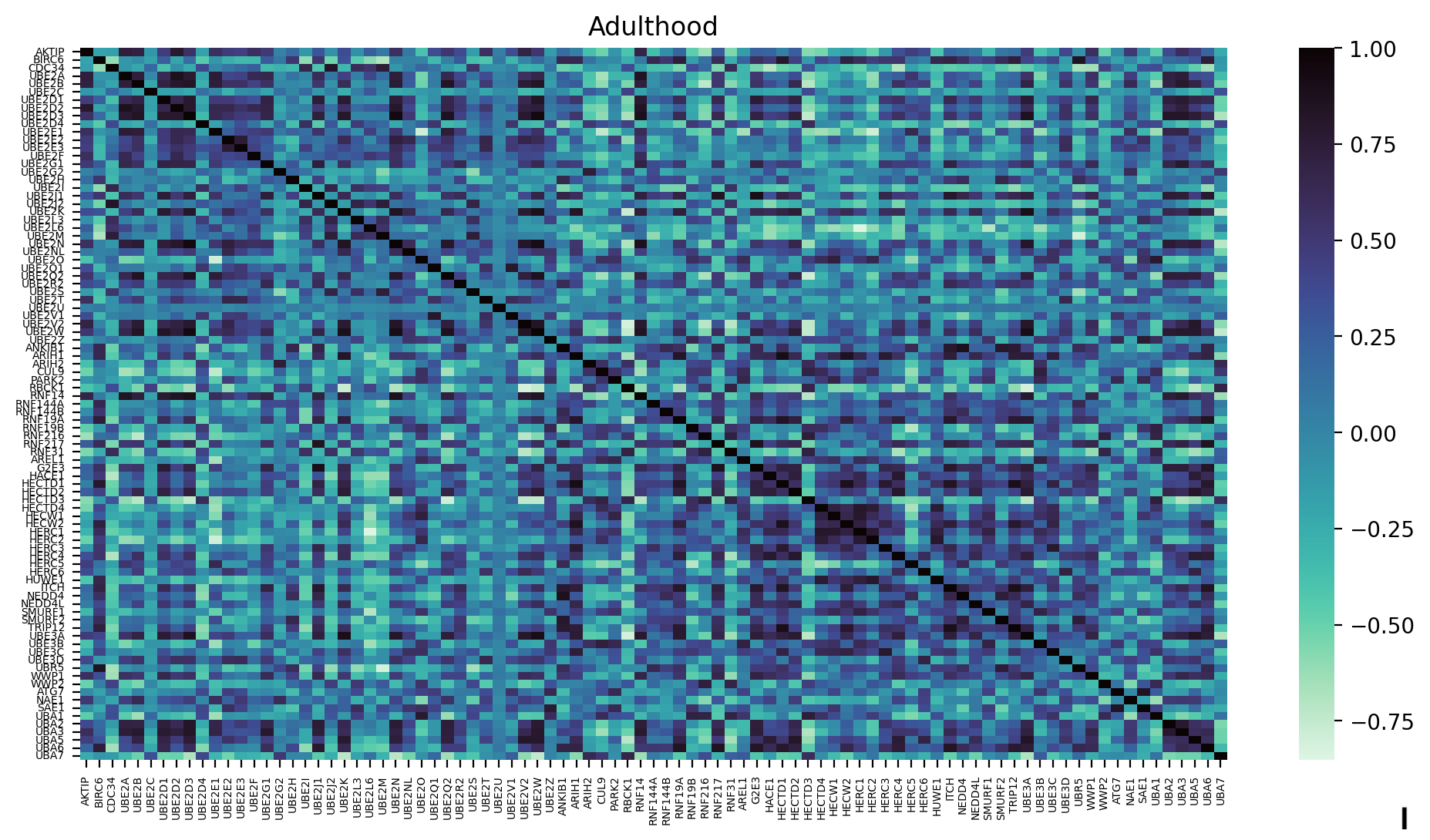

Supplement: Supplementary file 1 [file ijms-25-02361-s001.zip › Figure S1/I.png]

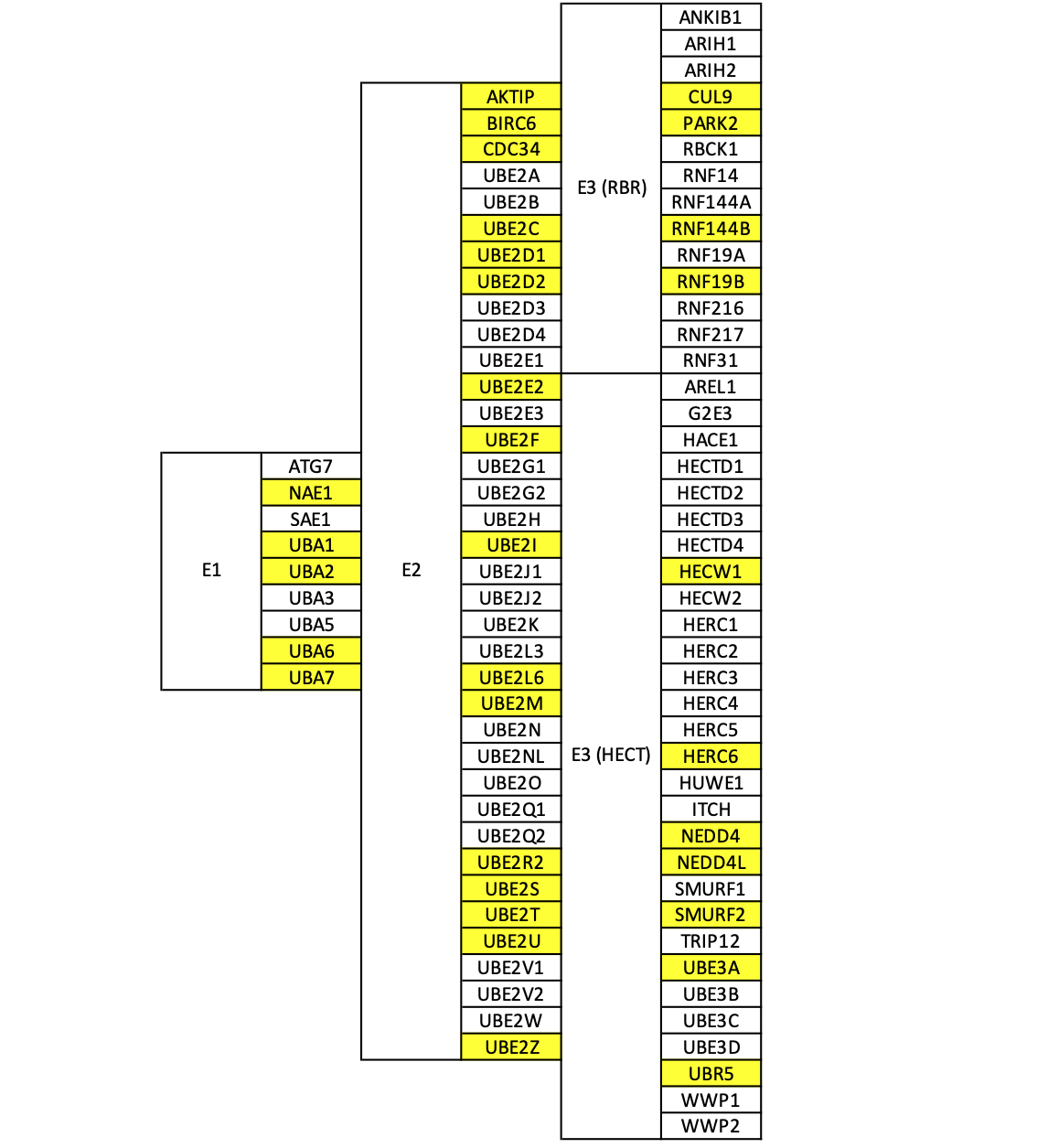

Supplement: Supplementary file 1 [file ijms-25-02361-s001.zip › Table S1 .png]
